# Physical training attenuates systemic cytokine response and tissue damage triggered by apical periodontitis

**DOI:** 10.1038/s41598-024-58384-1

**Published:** 2024-04-05

**Authors:** Railson de Oliveira Ferreira, Matheus Soares Pereira, Deiweson Souza-Monteiro, Deborah Ribeiro Frazão, João Daniel Mendonça de Moura, Daiane Claydes Baia-da-Silva, Leonardo Oliveira Bittencourt, Gabriela de Souza Balbinot, Fabrício Mezzomo Collares, Maria Laura de Souza Lima, Aurigena Antunes de Araújo, Rafael Rodrigues Lima

**Affiliations:** 1 Laboratory of Functional and Structural Biology, Institute of Biological Sciences, Federal University of Pará, Belém, Pará Brazil; 2 Dental Materials Laboratory, Department of Conservative Dentistry, School of Dentistry, Federal University of Rio Grande do Sul, Porto Alegre, Rio Grande do Sul Brazil; 3 Department of Biophysics and Pharmacology, Federal University of Rio Grande do Norte, Natal, Rio Grande do Norte Brazil

**Keywords:** Dental diseases, Oral diseases

## Abstract

Apical periodontitis (AP) is a condition characterized by inflammatory and infectious components in the tooth canal. AP affects periradicular tissues and has systemic repercussions. Physical exercise is a structured activity that requires cardiorespiratory function, and can modulate the inflammatory profile in pathological conditions. As a result, this study aimed to determine the effects of aerobic physical training (PT) on the alveolar bone with and without AP, and its systemic inflammatory repercussions. AP was induced in the mandibular first molars, and PT was performed on a treadmill for five consecutive days over four weeks, with progressive increases in speed and activity time. Blood samples were collected to determine serum cytokine levels using immunoassays, and alveolar bone samples were collected for histopathological evaluation, lesion volume and microarchitecture assessment using computed microtomography. Animals with AP had increased pro-inflammatory cytokines levels compared to those without AP; however, these levels were attenuated or restored by PT. Compared to the AP group, the AP + PT group had a smaller lesion volume and greater preservation of the bone trabeculae in the remaining alveolar bone surrounding the lesion. In overall, PT minimized the severity of AP proving to be a valid strategy for individuals undergoing endodontic treatment.

## Introduction

Physical activity is defined as any skeletal movement that requires more energy than that used by the body at rest^1^. Physical training differs from physical activity in terms of planning and structure, and considers the duration, frequency, and intensity of the activity^2^. Physical exercise promotes overall health by preventing and reducing inflammatory processes associated with various diseases^3^. Accordingly, several training modalities can stimulate biological systems, such as the musculoskeletal and cardiorespiratory systems, and different planning strategies are required to achieve the beneficial effects of physical exercise. Thus, to be considered a successful exercise program, moderate-intensity training should be recommended, as people inexperienced in high-intensity exercise may discontinue this activity. High-intensity training can also increase the risk of injury or acute cardiovascular events^4^.

The effects of physical training may be related to its involvement in inflammatory and immunological mechanisms^5^. Physical training modulates cytokines produced and released by the skeletal muscles, known as myokines, such as interleukin-6 and interleukin-8^6^. As a result, physical training has been proposed as a non-pharmacological strategy to reduce systemic inflammation^5^. A systematic review concluded that interleukin-6, tumor necrosis factor-α, interferon-γ, and C-reactive protein are key biomarkers of reduced inflammation caused by physical exercise in patients with atherosclerosis, a progressive and prevalent inflammatory disease^7^.

Apical periodontitis (AP) is another highly prevalent disease that causes an inflammatory reaction in the periapical tissues due to microbial infection in the dental pulp^8^. Infection occurs in the root canal after the pulp becomes necrotic due to caries, trauma, periodontal disease, or iatrogenesis, or when the pulp is absent due to previous root canal treatment. Once the infection is established in the root canal, it progresses in the apical direction, eventually reaching the periradicular tissues through the apical and lateral foramina, and promotes inflammation. AP is diagnosed, and depending on different bacterial- and host-related factors, this disease can be symptomatic (acute) or asymptomatic (chronic)^9^. AP advancement can promote serious alterations in the dental supporting apparatus as it progressively causes bone resorption, which can even culminate in tooth loss^10^. This severe pathogenic mechanism profile has significant implications in terms of aggression, as it is intrinsically related to systemic manifestations, such as the modulation of blood inflammatory markers^11^.

The levels of interleukin, C-reactive protein, and immunoglobulin are indicators of low-grade systemic inflammation caused by AP^11^. Cytokines participate in the degradation of extracellular matrix constituents and promote changes in periodontal cells. The exit of bacteria and their products from the apical foramen into periapical tissues triggers proinflammatory and anti-inflammatory mediators, including cytokines and other enzymes responsible for the activation of the inflammatory response. When the levels of inflammatory mediators reach a certain threshold, bone resorption pathways are activated, stimulating the action of fibroblasts and osteoblasts, which promote bone resorption^12^.

Endodontic infections can significantly influence the development and maintenance of systemic disorders, either through hematogenous dissemination of the microorganisms themselves, dissemination of their toxins, or induction of a low-intensity systemic inflammatory response^11^. Based on the benefits of physical training in modulating systemic features, this study aimed to determine the effects of physical training on rats with AP through an evaluation of both local and systemic repercussions.

## Results

### Evaluation of the systemic cytokine concentrations (IL-1β, TNF-α, IL-6, IL-10)

**Figure 1:**
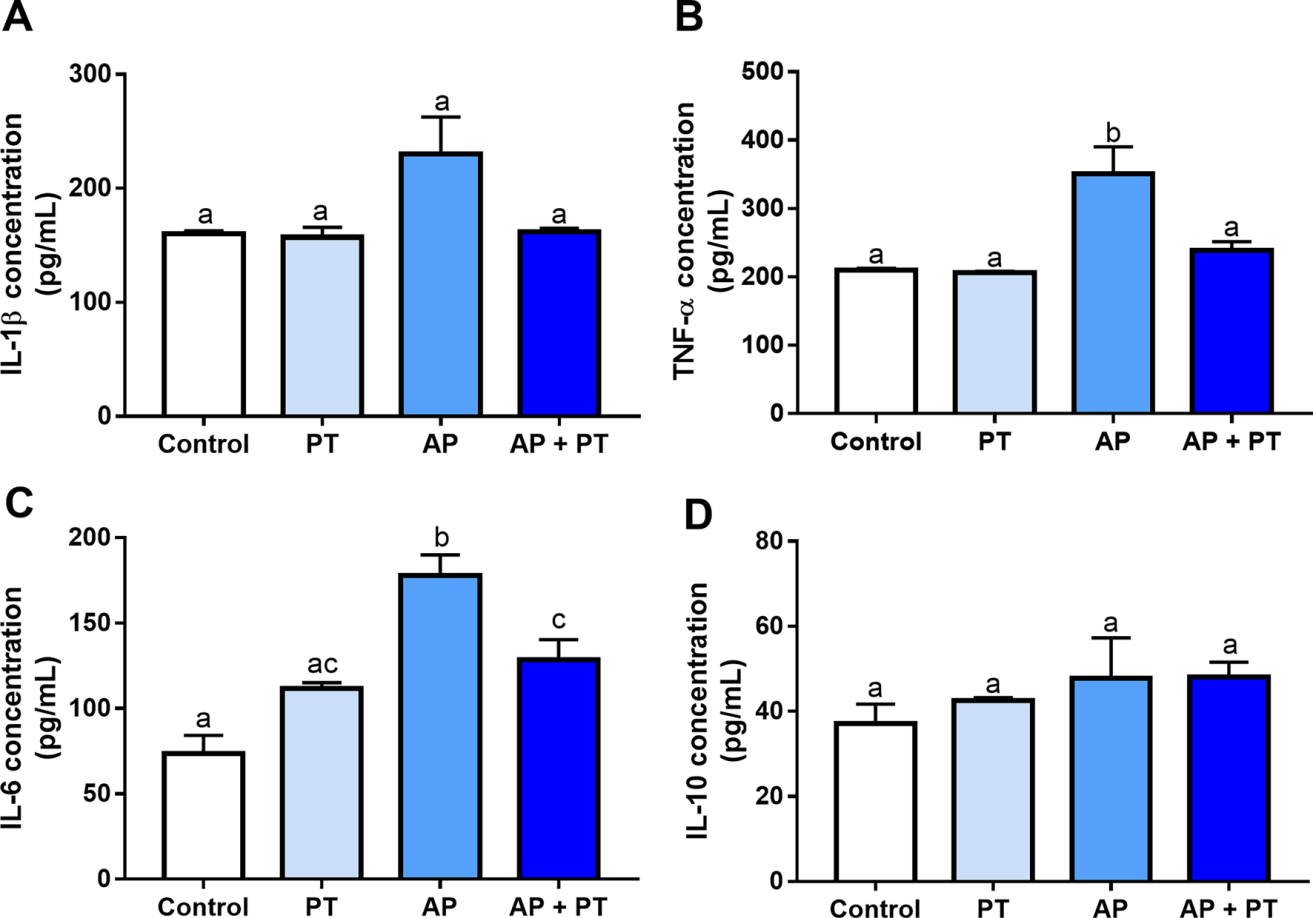
Plasma cytokine concentrations of physically trained animals with apical periodontitis. Plasma concentration of IL-1beta (**A**), TNF-alpha (**B**), IL-6 (**C**), and IL-10 (**D**). The results are expressed as pg/mL. Different letters indicate statistically significant difference (*p* < 0.05). The analysis was performed using one-way ANOVA with Tukey’s post hoc test (n = 8/*per* group).

Plasma levels of TNF-α (Fig. 1B) and IL-6 (Fig. 1C) were increased in the AP group compared to the control (adj. p value: 0.0063) and training (adj. p value: 0.0055) groups. When the animals with AP were subjected to physical training, the TNF-α concentration was reduced to values equivalent to those of the control (adj. *p* value: 0.777). Overall, AP increased the level of IL-6, which was significantly attenuated by physical training (adj. *p* = 0.0230). The levels of IL-1β and IL-10 did not change in any of the experimental groups.

### Physical training modulates the inflammatory response and minimizes the damages to the remaining alveolar bone

**Figure 2:**
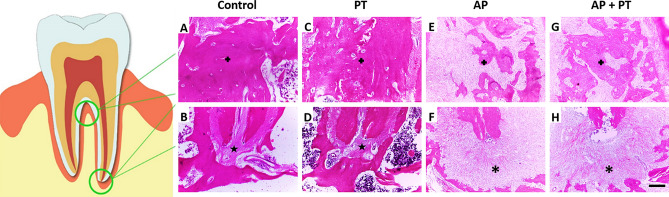
Implication of moderate-intensity physical training on inflammatory modulation in the furcation and apical regions of animals with apical periodontitis. 40 × magnification of the furcation and apical regions, respectively, of the maxillary first molar in the control (**A**, **B**), physical training (**C**, **D**), apical periodontitis only (**E**, **F**), and physically training + apical periodontitis (G, H) groups. Scale bar = 100 μm. In A, C, E, and D, the cross ( +) represents interradicular bone. In B and D, the star (✰) represents the periodontal ligament space. In F and H, the asterisk (*) represents the lesion area.

When the physically trained group with AP (G,H) was compared to the group with AP only (E,F), the interradicular bone was found to be more preserved in the furcation region of the physically trained group with AP,). Similar to the apical region, greater bone damage was identified in the group with AP (F) as the lesion area in this group was more extensive than that in the physically trained group with AP (H), which showed better preservation of the alveolar bone (Fig. 2), ultimately complementing and confirming the micro-CT results.

### Physical exercise minimizes bone damage in rats with AP

**Figure 3:**
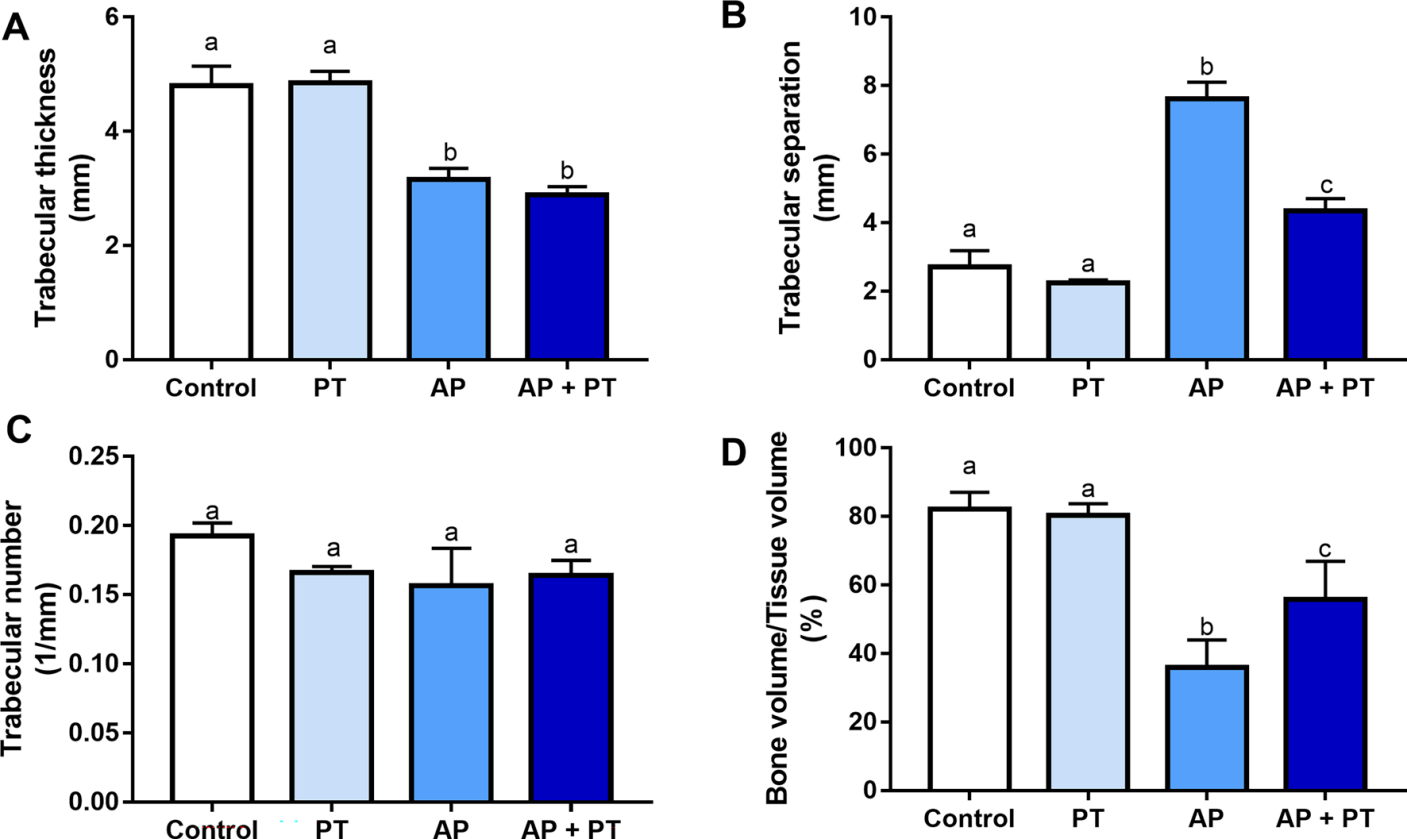
Effects of physical training on the alveolar bone of animals with apical periodontitis. Trabecular thickness in millimeters (**A**). Trabecular separation in millimeters (**B**). Trabecular number in 1/millimeters (**C**). Bone volume per tissue volume in percentage (**D**). The results are expressed as mean ± SEM. Different letters indicate a statistically significant difference (*p* < 0.05). The analysis was conducted using one-way ANOVA with Tukey’s post hoc test (n = 8/group).

The osteomodulatory effects of physical training on the remaining alveolar bone in rats with AP are shown in Fig. 3. Although no changes in trabecular thickness were observed beyond the expected thickness in the AP groups (*p* < 0.05; Fig. 3A), physical training could attenuate the impairment caused by AP over trabecular separation (adj. *p* value: < 0.0001; Fig. 3B).

The trabecular number did not change in any of the experimental groups (Fig. 3C); however, the proportions of bone volume and tissue volume were significantly attenuated by physical training in animals with AP (adjusted *p* value: 0.0042; Fig. 3D).

### Physical training reduces the volume of induced periapical lesion in rats

**Figure 4:**
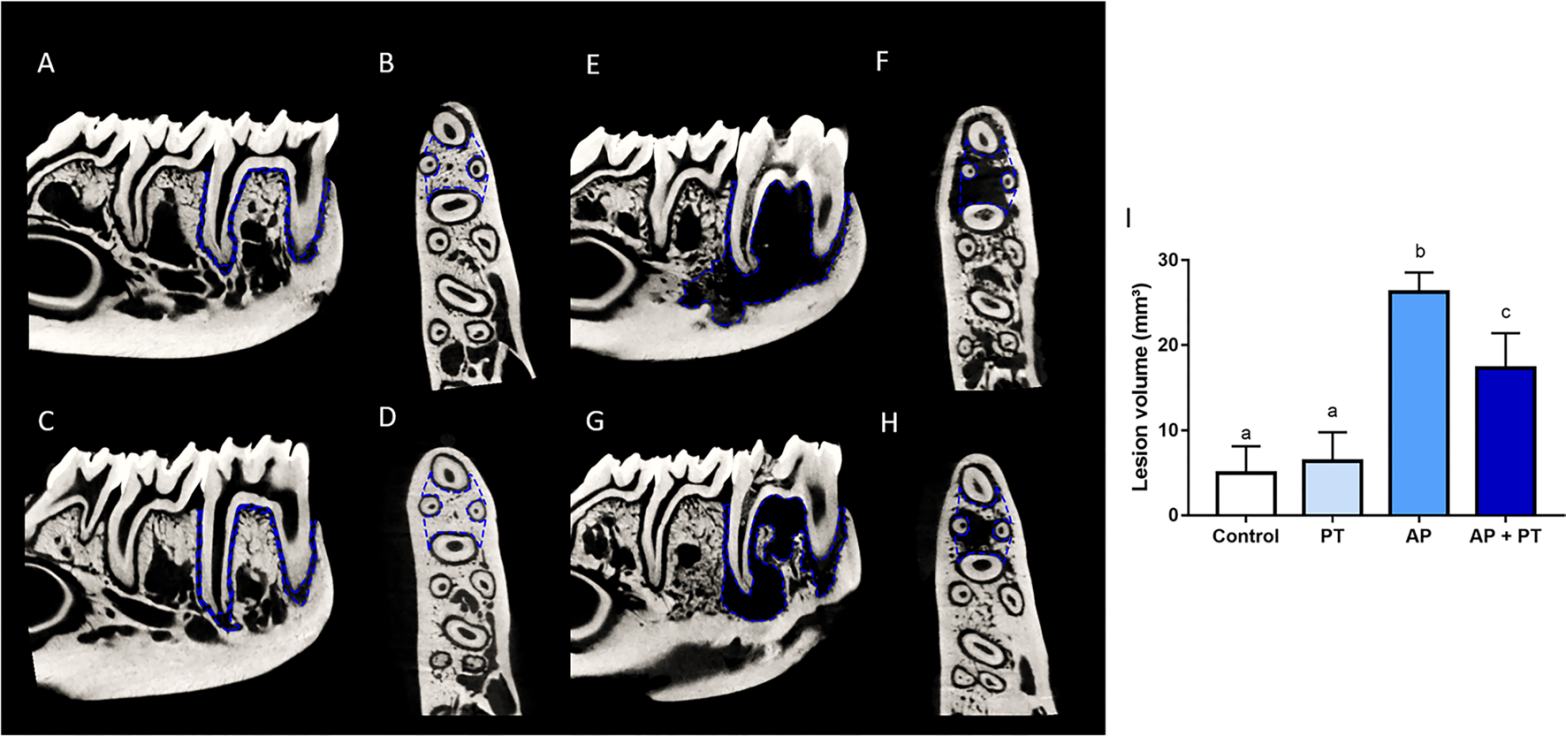
Effects of physical training on the lesion volume of animals with apical periodontitis. Representative sagittal and transverse slices of the hemimandibles of animals in the control (**A**, **B**, respectively), physical training (**C**, **D**, respectively), apical periodontitis (E, F, respectively), and apical periodontitis + physical training (G, H, respectively) groups. The blue dashed lines on the sagittal images represent the volume of interest used to measure lesion volume (mm3), and the blue dashed lines on the transverse images represent the region of interest for evaluating the quality of the remaining alveolar bone. Different letters indicate a statistically significant difference (*p* < 0.05). The analysis was performed using one-way ANOVA with Tukey’s post hoc test (n = 8/group).

Regarding apical periodontitis damage, the microtromographic assessment revealed a significant increase in lesion volume in sedentary animals compared to control and trained animals (Fig. 4; adj. *p*-value: < 0.0001). However, physical training attenuated the lesion volume compared to that found in lesioned and sedentary animals (adjusted *p*-value: 0.0029).

## Discussion

To the best of our knowledge, this is the first study to demonstrate that physical training comprising structured frequency and speed reduces the systemic inflammatory profile triggered by AP and attenuates the morphological damage to the alveolar bone of rats, including bone quality and periapical lesion area. This preclinical study provides important scientific data that are relevant in endodontics and sports dentistry, and highlights the beneficial effects of physical training in the minimization of AP-induced damage.

Different protocols are available for inducing AP in experimental models^13–18^. In this study, AP was induced by accessing the pulp chamber and exposing it to the oral environment, enabling infection and inflammation. This model has been well established in previous studies^14,15,19^. A 28-day experimental period is employed because periapical lesions actively expand for 3–4 weeks after pulpal exposure (active phase) and progress slowly thereafter (chronic phase)^20^.

AP can be either acute or chronic. Acute infections are caused by highly virulent bacteria or the synergistic effects of different species. Acute infections are characterized by a high concentration of bacteria that can invade tissues and reduce host resistance, while chronic AP is usually associated with less-virulent bacterial communities. AP contributes to low-grade systemic inflammation, leading to an overall increase in the levels of systemic inflammatory mediators^21^. This chronic inflammatory condition is associated with increased inflammatory cell infiltration and serum cytokine levels, which may affect systemic health^14^. Studies on animals with AP revealed an increase in the blood levels of TNF-α, IL-1β, IL-2, IL-6, IL-10, MMP-1, MMP-2, C-reactive protein (CRP), and immunoglobulin (IgA, IgG, and IgM)^11,14^. Thus, therapies and strategies that can minimize the high systemic inflammatory cytokine levels in this condition must be examined to ultimately reduce the predisposition to systemic diseases and progression of the lesion itself.

The cytokine network is a fundamental part of the immune/inflammatory response scenario of AP. In fact, the cytokine network plays an important role along with components of the immune system, vasoactive amines, and metabolites of arachidonic acid^10^. Plasma cytokine evaluation revealed alterations caused by both AP and physical training (Fig. 1). Animals with AP had higher TNF-α levels than control animals; however, animals with AP that underwent physical training showed a statistically significant decrease in the level of these proinflammatory markers compared to untrained animals with AP. The levels of TNF-α in the AP + PT group were also reduced to levels similar to those found in animals without AP (control or physical training only). TNF-α produces a wide range of effects on different cell types, thereby affecting immune and proinflammatory responses^22^. TNF-α and interleukins are involved in periapical pathogenesis related to bone resorption mechanisms^23,24^. A study revealed bone extracellular matrix destruction by stimulating the proteolytic activity of plasminogen and activating osteoclasts due to the presence of TNF-α^23^. The involvement of TNF-α in bone destruction caused by AP may explain the relation between the reduced levels of this cytokine and the minimization of bone destruction after physical activity.

Lesion volume and bone quality were evaluated using micro-CT, a non-destructive method that enables an accurate three-dimensional evaluation of the cortical and trabecular bone^25,26^. Notably, the alveolar bone microstructure in rats differs from that of other bones owing to its neuroectodermal origin. In fact, the alveolar bone microstructure consists of compact bone with highly mineralized plate-like trabeculae, limited bone marrow, large trabecular thickness, and reduced number of trabeculae and trabecular distances. Changes in these alveolar bone microstructures can be caused by AP, resulting in the resorption of the apical region, which increases the chances of bone destruction^13^. Based on our findings, animals with AP had alterations in the alveolar bone structure compared to animals without AP. In particular, AP increased the distance between the trabeculae and decreased the trabecular thickness and bone volume (Fig. 3). According to the literature, AP modulates such changes in the alveolar bone of rats mainly due to alterations in bone metabolism caused by cytokines and pathway activation in the blood supply^27^.

Interestingly, the AP-induced damage to the alveolar bone structure might be reduced by physical activity. The trained animals with AP had a reduced distance between the trabeculae and a higher percentage of bone volume than sedentary animals with AP. Physical training may modulate bone metabolism by attenuating the harmful effects exerted on its microstructure. Furthermore, the lesion volume due to AP was reduced after physical training. Reduced lesion volume is an remarkable finding that can provide suggestions for the modulation of AP pathogenesis by physical training. The smaller dimensions of the lesions in the trained animals are shown in Fig. 4. A smaller lesion can attenuate bone damage and systemic repercussion caused by AP and is associated with a better prognosis^28^. These volume proportions suggest that the alveolar bone of trained rats has a microarchitecture that is more resistant to lesion progression and may be less affected by the inflammatory immune response.

IL-6 is another cytokine involved in AP. The production of IL-6 occurs after the secretion of TNF-α and IL-1. Thereafter, IL-6 may inhibit the liberation of these cytokines, assuming control over vascular permeability and osteoclast differentiation^29^. Overproduction of IL-6 has been found to be related to the activity of macrophages and neutrophils present in the first stages of periapical lesion formation^29^. Prior studies have suggested a correlation between high IL-6 levels and larger lesion volume^30^. Based on our findings, physical training reduced IL-6 levels in animals with AP. This modulatory effect of physical exercise on IL-6 highlights the magnification repercussions of the lesion volume. The levels of IL-6 may be associated with a different remodeling process, which could explain our micro-Ct findings.

In accordance with the microtomography findings, histopathological analysis revealed greater preservation of the remaining alveolar bone in the AP + PT group than that in the AP group. Periapical lesions are histologically characterized by fibrous and granulated tissues infiltrated by different inflammatory cells^31^. Histopathological photomicrographs revealed inflammatory infiltrates in the lesion area. An in vivo study comprising a histological analysis revealed that physical exercise had beneficial effects on bone remodeling in diabetic rats^32^.

Low-grade systemic inflammation promoted by AP leads to an imbalance in the mechanisms involved in the modulation of serum cytokine levels, mainly related to a decrease in the activity of regulatory T lymphocytes (Tregs)^24^. Tregs are a subpopulation of T-cells responsible for suppressing T-cell responses and limiting disease progression. In human periodontitis, Tregs can express TNF- α, which might regulate the immunologic process of periodontitis. IL-6 negatively regulates T cell differentiation, the inflammatory response, and osteoclast differentiation^33^. Thus, the role of physical activity and exercise modulation in cytokine imbalance may be related to periodontitis and a reduction in periapical lesion progression^34^.

The effects of physical training on bone tissue have garnered attention in studies related to sports and systemic diseases with bone implications, such as osteoporosis, rheumatoid arthritis, and obesity^5^. Physical activity maximizes bone mass, increases bone resistance, and decreases fracture risk^35,36^. Exercise affects bone remodulation and improves mineralization and geometry^35^. However, information and a gold standard for the beneficial effects of physical straining on the modulation of inflammatory responses are lacking^37^.

The findings of this study are promising owing to increased interest in research on the effects of physical activity as a modulator of systemic conditions caused by many inflammatory diseases. Our findings are relevant to the field of sports dentistry, highlighting the need for more studies and the publication of consistent literature on the issue. This study had some limitations. Our results should be extrapolated with caution and new studies are required to better understand the mechanisms and relationships between physical training and AP. Our study revealed the protective effect of physical training during disease progression. Notably, the effects of the chronological occurrence of events could be an important future assessment. New studies could reveal the behavior of AP in individuals with a previous history of physical training and evaluate the local and systemic effects of physical training in individuals after the development of AP.

## Conclusion

Physical activity can reduce the damage caused by AP, thereby modifying systemic levels of TNF-α and IL-6, and leading to the minimization of local damages. The changes in the periapical bone based on micro-CT and histological analyses reflected a decrease in bone damage. Therefore, these results support the positive benefits of physical training on inflammatory processes, such as those reported in our AP model. However, further research is needed to elucidate the underlying molecular pathways and evaluate the relevant tissues.

## Methods

### Ethical aspects and formation of the experimental groups

This study was approved by the Ethics Committee on Experimental Animals of the Federal University of Pará (license number: 4090250320) in accordance with the Guide for the Care and Use of Laboratory Animals and Animal Research: Reporting of In Vivo Experiments (ARRIVE)^38^. The sample size was calculated using GPower 3.1.9.4 software based on a previous study^39^.

Thirty-two male *Rattus norvegicus* Wistar rats (age, 90 days old; weight, ~ 150 g) were obtained from the central vivarium of the Federal University of Pará. The animals were distributed in plastic cages (4 animals each), administered controlled food (Nuvital Nutrientes S/A, Paraná, Brazil) and filtered water ad libitum, and stored in a room under a 12-h light/dark cycle and controlled temperature (25 ± 1 °C).

**Figure 5:**
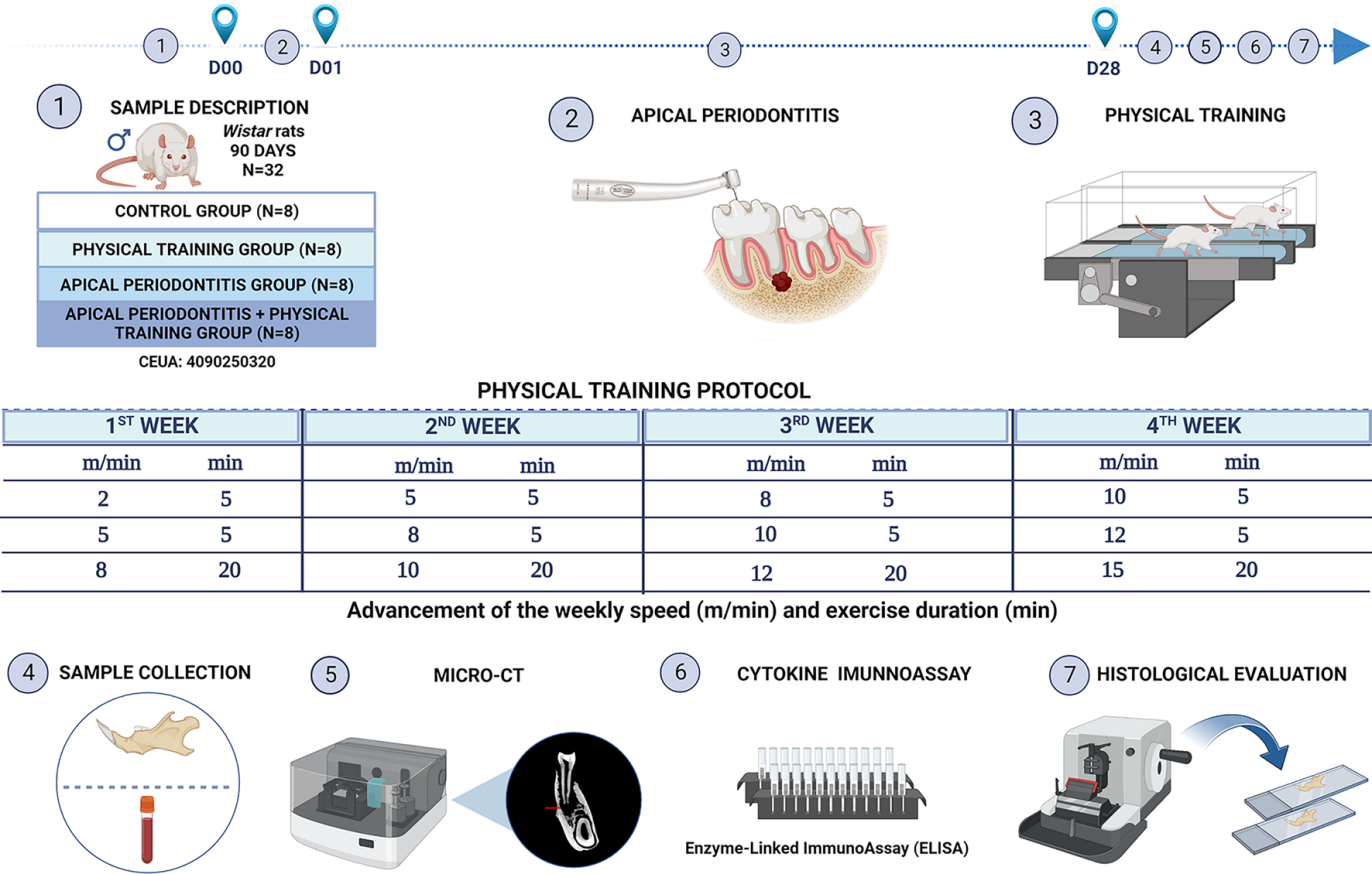
Description of the methodology. (1) Sample and experimental group descriptions. (2) Induction of apical periodontitis. (3) Physical training protocol. (4) Collection of the biological samples. (5) Measurement of cytokine levels using enzyme-linked immunoassay. (6) Histological evaluation. (7) Assessment of alveolar bone effects using computed microtomography.

The animals were divided into four experimental groups (8/*per* group): control (sedentary animals), AP, physical training (PT), and AP plus physical training (AP + PT). The experimental design is illustrated in Fig. 5.

### Apical periodontitis induction

One day before the beginning of the physical training protocol, animals in the AP and AP + PT groups were anesthetized with intraperitoneal injections of 9 mg/kg 2% xylazine hydrochloride (2 mg/mL) and 90 mg/kg 10% ketamine hydrochloride (10 mg/mL). After anesthesia, the pulp chamber was accessed in the distal fossa of the mandibular first molars using a #1/4 low-speed carbide bur. The pulp chamber was left open for 28 days to enable development of the AP lesions. This method is widely used and accepted according to the literature. Periapical lesions can be established within the required timeframe using this technique^9^. A daily dosage of buprenorphine (0.05 mg/kg on the day of the procedure and 0.1 mg/kg for the following 2 days). This medication was administered to alleviate pain and discomfort associated with the surgical procedure, including pulp exposure.

### Physical training

A motorized treadmill designed for rodents (50 cm long and 10 cm wide) with lanes divided into acrylic walls was employed for the physical training program. The running sessions lasted 30 min each training session. Five sessions were held each week (one session per day) for four consecutive weeks.

This protocol, which comprises exercise intensity (60% VO_2_ max), was based on the parameters used in previous studies^39,40^. Each training session began with a 10-min warm-up at a slower speed, followed by a weekly speed schedule. Each week, the distance and running speed were gradually increased (Fig. 1).

### Perfusion and sample collection

Animals were anesthetized as previously described. Blood was collected and centrifuged at 3.000 rpm, and the obtained serum was collected and stored in an ultrafreezer at −80 °C for further analysis. The animals were perfused through the left ventricle of the heart with 0.9% saline solution, followed by 4% formaldehyde. The hemimandibles were collected and stored in 4% formaldehyde solution. The left hemimandibles were used for microtomographic analysis, whereas the right hemimandibles were used for histological analysis.

### Evaluation of the serum cytokine concentrations

Serum samples (n = 8/group) were analyzed as described previously^42^. Briefly, the levels of interleukin-1beta (IL-1β) (detection range: 62.5–4000 pg/mL; minimum detection limit: 12.5 ng/mL), interleukin-6 (IL-6) (detection range: 125–8000 pg/mL), interleukin-10 (IL-10) (detection range: 62.5–4000 pg/mL; minimum detection limit: 12.5 ng/mL), and tumor necrosis factor-alpha (TNF-α) (detection range: 62.5–4000 pg/mL; minimum detection limit: 50 ng/mL) were determined using commercial enzyme-linked immunosorbent assay kits (R & D Systems, Minneapolis, MN), according to the manufacturer's instructions. The results are expressed as pg/mL.

### Histological evaluation

The right hemimandibles were rinsed under running water for 4 h and then demineralized in 10% ethylenediaminetetraacetic acid (EDTA) for 90 days with weekly solution changes. After demineralization, the samples were rinsed under running water for 4 h. The specimens were dehydrated with alcohol, cleared in xylene, and embedded in paraffin. Sections of 5 μm thickness were cut using a Leica RM 2045 microtome (Leica Microsystems, Nussloch, Germany) in the mesiodistal orientation and mounted on individual slides. The sections were stained with hematoxylin and eosin, and photomicrographed using a digital camera (DS-Fi3; Nikon, Tokyo, Japan) attached to a microscope (Nikon Eclipse CiH550s; Tokyo, Japan). Images were subjected to histopathological analyses.

### Bone analysis using computerized microtomography (Micro-CT)

The left hemimandibles were scanned using X-ray micro-CT (MicroCT.SMX-90 CT; Shimadzu Corp., Kyoto, Japan). Images were obtained with a 360° rotation and an intensity of 70 kV and 100 mA. Thereafter, the images were reconstructed using the inspeXio SMX-90CT software (Shimadzu Corp., Kyoto, Japan) with a voxel size of 10 µm, thickness of 14 µm, and resolution of 1024 × 1024, producing 536 photos per sample.

Alveolar bone resorption and alveolar bone tissue quality were analyzed using CTAn software (V1.15.4.0; Bruker, Kontich, Belgium). The alveolar bone resorption area was analyzed as described previously^43^. The reconstructed volume of interest (VOI) in the sagittal plane was determined, including the periodontal ligament space and destruction around the root of the mandibular first molars. The VOI was determined from the first coronal cut of the mesial root of the mandibular first molar to the last cut in the distal region and ended upon reaching the mandibular second molar.

The quality of the remaining alveolar bone that was unaffected by the lesion was evaluated using a set of approximately 150 images surrounding the roots of the mandibular first molar. The region of interest (ROI) was determined in the transverse plane from the point closest to the mesial root to the point farthest from the distal root. Segmentation was performed on the images with grayscale thresholds ranging from 120 to 225. The following parameters were measured: trabecular thickness (Tb.Th), trabecular spacing (Tb.Sp), trabecular number (Tb.N), and bone volume percentage (BV/TV).

### Statistical analyses

Statistical analyses were performed using GraphPad Prism 7.0 software (GraphPad Software Inc., La Jolla, CA, USA). The Shapiro–Wilk test was conducted to assess normality. For comparisons between groups, one-way ANOVA followed by Tukey’s post-hoc test was performed. A *p* value < 0.05 was considered to indicate statistical significance. The results are presented as mean ± standard error of the mean (SEM). All the values of our analysis are available on the Supplementary Table S1.

### Supplementary Information

Supplementary Information.

## Data Availability

All the data analyzed in this study is included in this article and in the supplementary table S1.
