# Supplementary material for: Physical training attenuates systemic cytokine response and tissue damage triggered by apical periodontitis
Source: Sci Rep. 2024 Apr 5;14:8030. doi: 10.1038/s41598-024-58384-1 (PMC10997662; doi:10.1038/s41598-024-58384-1)
Supplement: Supplementary file 1 — Supplementary Information. [file 41598_2024_58384_MOESM1_ESM.pdf]

Physical training attenuates systemic cytokine response and tissue damage triggered by apical periodontitis

Railson de Oliveira Ferreira[1+], Matheus Soares Pereira[1+] Deiweson Souza-Monteiro[1], Deborah Ribeiro Frazão[1], João Daniel Mendonça de Moura[1], Daiane Claydes Baia-da-Silva[1], Leonardo Oliveira Bittencourt[1], Gabriela de Souza Balbinot[2], Fabrício Mezzomo Collares [2], Maria Laura de Souza Lima[3], Aurigena Antunes de Araújo[3], and Rafael Rodrigues Lima[1*]

[1] Laboratory of Functional and Structural Biology, Institute of Biological Sciences, Federal University of Pará, Belém, Pará. Brazil
[2] Dental Materials Laboratory, Department of Conservative Dentistry, School of Dentistry, Federal University of Rio Grande do Sul, Porto Alegre, Rio Grande do Sul, Brazil
[3] Department of Biophysics and Pharmacology, Federal University of Rio Grande do Norte, Natal, Rio Grande do Norte, Brazil

[*] rafalima@ufpa.br
[+]these authors contributed equally to this work

Table S1. Description of all analysis values of the study.

Analysis	Description of unit	Mean (Control)	SD (Control)	SEM (Control)	Mean (Physical Training)	SD (Physical Training)	SEM (Physical Training)	Mean (Apical Periodontitis)	SD (Apical Periodontitis)	SEM (Apical Periodontitis)	Mean (Apical Periodontitis + Physical Training)	SD (Apical Periodontitis + Physical Training)	SEM (Apical Periodontitis + Physical Training)
**IL-1β**	pg/mL	37.08	8.03	4.64	42.50	1.25	0.72	47.80	16.50	9.52	48.02	6.09	3.51
**TNF-α**	pg/mL	210	5.000	2.887	206.7	2.887	1.667	350.7	68.79	39.72	238.3	22.55	13.02
**IL-6**	pg/mL	73.33	18.93	10.93	111.7	5.77	3.33	177.9	29.38	11.99	128.3	29.10	11.88
**IL-10**	pg/mL	160	5.00	2.88	157.2	14.95	8.63	230.5	55.56	32.08	161.7	5.77	3.33
**Tb.Th**	mm	4.97	1.15	0.34	4.85	0.67	0.20	3.16	0.57	0.19	2.88	0.46	0.14
**Tb.Sp**	mm	2.71	1.51	0.47	2.24	0.27	0.086	7.61	1.39	0.49	4.34	1.01	0.35
**Tb.N**	1/mm	0.19	0.026	0.009	0.16	0.004	0.01	0.15	0.02	0.065	0.16	0.01	0.034
**BV/TV**	%	82.23	4.79	2.39	80.30	3.43	1.53	35.91	8.02	3.58	55.82	11.02	4.92
**Lesion volume**	mm^3	4.99	3.15	1.41	6.39	3.40	1.52	26.27	2.28	1.02	17.31	4.10	1.835

Table S1: Values of the experimental study: systemic inflammatory biomarkers evaluation (IL-1β: Interleukin-1β, TNF-α: Tumoral Necrosis Factor-α, IL-6: Interleukin-6, IL-10: Interleukin-10) and microtomographic analysis (Tb.Th: Trabecular thickness, Tb.Sp: Trabecular spacing, Tb.N: Trabecular number, BV/TV: ratio between bone volume and tissue volume and Lesion volume). Results are expressed as mean, SD: Standard deviation and SEM: Standard error of mean.
